# Postcesarean Thromboprophylaxis with Two Different Regimens of Bemiparin

**DOI:** 10.1155/2011/548327

**Published:** 2011-12-26

**Authors:** Milagros Cruz, Ana M. Fernández-Alonso, Isabel Rodríguez, Loreto Garrigosa, Africa Caño, Pilar Carretero, Amelia Vizcaíno, Amanda Rocío Gonzalez-Ramirez

**Affiliations:** ^1^Obstetrics and Gynecology Department, San Cecilio University Hospital, Avenida Madrid No. 16, 18012 Granada, Spain; ^2^Obstetrics and Gynecology Department, School of Medicine, University of Granada, Spain; ^3^Methodology and Statistics Department, FIBAO and San Cecilio University Hospital, Granada, Spain

## Abstract

*Objectives*. To compare the effectiveness of postcesarean thromboprophylaxis with two different regimens of bemiparin. *Material and Methods*. The study included 646 women with cesarean delivery in our hospital within a 1-year period, randomly assigned to one of two groups for prophylaxis with 3500 IU bemiparin once daily for 5 days or 3500 IU bemiparin once daily for 10 days. *Results*. There was one case of pulmonary embolism (first day following cesarean). An additional risk factor was present in 98.52% of the women, most frequently emergency cesarean, anemia, or obesity. The only risk factors for thromboembolic disease significantly related to pulmonary thromboembolism were placental abruption and prematurity. There were no differences in thromboembolic events among the two thromboprophylaxis regimens. *Conclusions*. Cesarean-related thromboembolic events were reduced in our study population due to the thromboprophylactic measures taken. Thromboprophylaxis with 3500 IU bemiparin once daily for 5 days following cesarean was sufficient to avoid thromboembolic events.

## 1. Introduction

Venous thromboembolism (VTE) remains one of the main direct causes of maternal mortality in developed countries [1–4], largely due to pulmonary thromboembolism (PE) [5–7] which is responsible for around 20% of maternal deaths [8]. Epidemiologic studies estimate the annual frequency of deep venous thrombosis (DVT) in the general population to be from 0.16‰ [7] to 1‰ [9], of which 2% are pregnancy related [5]. There is an increased risk of thromboembolic event, either DVT or PE, during pregnancy and puerperium [5, 10], and it has been estimated that the risk of VTE is 10-fold higher [11–15], reaching up to 2‰ [16]. 

Puerperium is the period with highest VTE risk [12–14, 17, 18] which was reported to be up to 25-fold higher than that in nonpregnant women [12–14]. It has been reported that 43–60% of pregnancy-related PE episodes take place during puerperium [11, 13].

The incidence of pregnancy-related VTE is estimated at 0.76 to 1.72‰ [11, 18].

The incidence is likely to be underestimated, since women are often asymptomatic [19] or present nonspecific signs or symptoms [20], and VTE during puerperium is often diagnosed or treated in a different hospital from where the delivery took place [20, 21].

The incidence of puerperium-related VTE is 0.65‰ [11].

Established risk factors for VTE during pregnancy include [9, 22] maternal age (1/800 for age >35 years; 1/1600 for age <35 years) [12, 13, 18, 23], obesity (body mass index (BMI) >30) [24, 25], preeclampsia/hypertension, parity ≥3 [16], previous VTE or congenital or acquired thrombophilia [17, 19, 20, 23, 26], smoking, diabetes [4], multiple gestation [25], black race [27], and anaemia. During the labour there are other factors [9]: type of delivery (with 3–6-fold higher risk for cesarean *versus* vaginal delivery, higher for emergency cesarean [13, 28, 29], and mid-cavity instrumental delivery) [28, 29], prolonged labour >12 hours [10, 25, 28], immobility; major abdominal surgery for >30 minutes during pregnancy or puerperium [12], preterm delivery [11], excessive blood loss (>1 litre), or blood transfusion. In the postpartum period, other factors may be added as dehydration, immobility, and anaemia [9, 22].

The factors that contribute the most to the incidence of thromboembolic events, due to their high prevalence, are age >35 years, obesity, and cesarean delivery [28].

Cesarean delivery increases the risk of VTE because it involves pelvic surgery that may last >30 minutes, adding to the prothrombotic effects of delivery, pregnancy weight gain, and other risk factors (see above).

The VTE incidence rate following caesarean section is 1.78‰ [11], with an odds ratio of 2 [11].

The latest guidelines [9, 16, 22] recommend that thromboprophylaxis with low-molecular-weight heparin (LMWH) in women who undergo an emergency cesarean and in women who undergo an elective cesarean also has an additional risk factor.

However, the duration of postcesarean thromboprophylaxis has been discussed and is an important factor, given the elevated risk of VTE during puerperium, especially during the first week postpartum [9, 22] and the trend to earlier postcesarean hospital discharge. So the latest guidelines [9] recommend thromboprophylaxis in these women during seven days.

However, there is no scientific evidence about the duration of the thromboprophylaxis, only clinical recommendations [7, 9, 22].

We investigated the incidence of VTE in women after cesarean section (who had not required LMWH during pregnancy) who received two different LMWH regimens in order to determine the best one to prevent this disease.

In our hospital, pneumatic compression is not used as a method of thromboprophylaxis. After using different LMWH (enoxaparin, nadroparina,…) as pharmacological thromboprophylaxis, the hospital finally established a policy with bemiparin as postoperative thromboprophylaxis (gastrointestinal, thoracic, gynaecological, obstetrics, urological, orthopedic surgery,…) several years ago.

The safety and efficacy of the LMWH bemiparin have been demonstrated for the treatment and prophylaxis of venous thromboembolism. Bemiparin is a second-generation LMWH. It is a sodium salt obtained by depolymerization of nonfractioned heparin derived from pig intestinal mucosa. Bemiparin has the lowest molecular weight (3600 Da), the longest half-life (5.3 h), and the highest anti-FXa/anti-FIIa activity ratio (8 : 1) of any second-generation LMWH [30].

The main objective was to compare the effectiveness of postcesarean thromboprophylaxis with two different regimens of bemiparin: 3500 IU once daily during five days *versus *ten days.

## 2. Material and Methods

This research was approved by the Research Ethical Committee of our university hospital.

### 2.1. Study

Comparison between five-day and ten-day bemiparin regimens (3500 IU once daily) as postcesarean thromboprophylaxis.

Inclusion criteria for this study were women with a cesarean delivery who had not required prophylaxis or treatment with any type of LMWH during pregnancy (low risk of VTE during pregnancy) and absence of allergy to heparin or derivatives. Later, those women who did not fulfill the duration of proposed prevention were excluded.

Outcome variables were the number of DVT and PE episodes and VTE-related maternal death up to 3 months following cesarean. Variables assessed as possible risk factors for a thromboembolic event were age, smoking, obesity (BMI > 30), hypertension, parity, multiple pregnancy, diabetes, week of delivery, type of cesarean (emergency or elective), type of anesthesia, blood loss, and immobility (at least three days).

During the period of study, in our hospital 2924 deliveries were attended; 742 cesarean were realized. All VTE cases were registered.

96 women who underwent a cesarean section were excluded because they did not fulfill the inclusion criteria.

The study included 646 women with cesarean delivery in our hospital during a 1-year period, assigned in a randomly systematic way to one of two groups for prophylaxis with 3500 IU bemiparin once daily for 5 days or 3500 IU bemiparin once daily for 10 days.

Bemiparin was administered at least 8 hours following cesarean. In case of locoregional anesthesia, Bemiparin was not given until 8 hours after removing the epidural catheter.

A complete blood count test was done to all women who underwent a cesarean within the next 24–36 hours.

We have chosen 5 and 10 days of thromboprophylaxis, because 5 days is recommended by the clinical practice guidelines before 2008, and we have chosen the comparison with 10 days, because the different clotting factors begin to normalize after the first week postpartum, although the risk may persist until the sixth week postpartum.

### 2.2. Statistical Analysis

Descriptive statistics were calculated for all study variables. The chi-square test was used to compare between 5-day and 10-day bemiparin.

A logistic regression model was used to analyze the demographic and morphometric variables and likelihood of DVT/PE (dependent variables) with each regimen.

## 3. Results and Discussion

### 3.1. Results

During this period of time, 646 women with cesarean delivery met the study inclusion criteria.  Table 1 shows the characteristics of the women and their possible thromboembolic risk factors during their pregnancy.

Mean age was 31 ± 5.47 years, 59.7% were nulliparous, 9% had ≥2 previous deliveries, and 14% were smokers. BMI indicated that 42.3% of patients in the bemiparin groups were obese (BMI > 30). Other possible risk factors for VTE were diabetes (0.8–4.82%), hypertension during pregnancy (10.7%), and multiple pregnancy (*≈*5%). There was a negligible incidence of prolonged immobility, drug consumption, or heart disease in our study population.

Table 2 shows the characteristics of the deliveries and the associated thromboembolism risk factors. Cesareans were almost always at term, with only 12.7% preterm, while 73.6% were emergency cesareans, an added thrombosis risk factor. The most frequent anesthesia was locoregional (88.3%). Other potential thrombosis factors (due to the possible underlying disease) were placental abruption (2.2%) and intrauterine growth retardation (IUGR) (8.5%). In the present study, only placental abruption and gestational age (preterm) were significantly related to PE (*P* < 0.005).

Table 3 depicts the thrombosis risk factors recorded after the cesarean. Anemia (Hb < 11) was the most prevalent risk factor (75.4% women after cesarean section), followed by hypertension (4.9%) and infection (6.4%). Almost all women (98.53%) had an additional risk factor beside the cesarean.

There were neither maternal deaths nor any DVT cases.

There was only one PE case. This case was in a 25-year-old nulliparous woman assigned to group B with a BMI of 29.5 and no family history of thrombosis; her smoking habit of <10 cigarettes/day was the only risk factor during pregnancy. She was hospitalized in week 32 with placental abruption symptoms and underwent emergency cesarean with locoregional anesthesia. At this time two other risk factors were added up: emergency cesarean section and vaginal bleeding. The fetus was delivered alive with appropriate weight for gestational age. The mother presented with moderate anemia that required intravenous iron treatment. On the first postoperative day, before beginning the thrombophylaxis with bemiparin, she reported difficulty in breathing and pain in right costodiaphragmatic recess. PE was diagnosed after clinical examination, elevated D-dimer, and complementary tests: perfusion lung scintigraphy (PE in lateral and medial segments of medial lobe of right lung) and bilateral lower limb Doppler's ultrasound examination (normal). Consequently, a therapeutic LMWH regimen was started instead of the thromboprophylaxis regimen and was subsequently replaced with oral anticoagulant therapy. A hypercoagulation test at 6 months ruled out thrombophilias.

Hence, the incidence of VTE in this series (women with cesarean delivery) was 1,54% and the incidence of VTE in total number of deliveries was 0,34% (1/2924).

Considering that the only PE case occurred on the first postoperative day, before beginning the thromboprophylaxis, comparisons among the different LMWH regimens were not appropriate.

### 3.2. Discussion

There is a continual and exhaustive surveillance of VTE risk factors during pregnancy and puerperium at our hospital, and appropriate thromboprophylactic measures are implemented when they are detected [31].

Those measures are recommendations of exercises for early mobilization for foot and legs, early ambulation (within two hours after the vaginal delivery and 6–8 hours after cesarean section), women are well hydrated with serum therapy in puerperium, and liquid and food are administered early (immediately after vaginal delivery; at 4–6 hours after cesarean section with locoregional anesthesia and 6–8 hours after cesarean section with general anesthesia) and LMWH thromboprophylaxis starts at least 8 hours following cesarean.

This policy may explain why only one episode of VTE was observed in the hospital during the period studied; this case took place during the first 24 hours after cesarean for placental abruption.

Given the total number of deliveries at the hospital (2924) during this period, an incidence from 2 to 5 VTE cases related to pregnancy, delivery, and puerperium could be expected [10, 11, 28].

The fact that only one case was observed may suggest a lower presence of risk factors in our population; nevertheless, 98.53% of studied women had at least one other thrombosis risk factor besides the cesarean surgery.

When we analyzed the risk factors, compared with that reported in other studies, we observed that, although women in our study have risk factors for VTE (age over 35 years 30%, obesity 30%, emergency cesarean section >70%, anaemia 50%). We only had one case of VTE (risk factors presented: smoking, emergency cesarean section, preterm delivery, vaginal bleeding, and severe anemia).

The most frequent risk factors in our population were emergency cesarean, anaemia, and obesity. Special attention is given to VTE prevention at our center, and all women are closely followed up for risk factors throughout their pregnancy and puerperium to determine any need for thromboprophylaxis.

In our hospital, the thromboprophylaxis measures may help to explain our finding of a lower-incidence VTE (0,34%) than that of in published reports [11, 32]. Regional anesthesia (epidural/dural) was used in more than 90%, cases of caesarean section, and this fact can reduce the risk of VTE [24].

Historically in our hospital, LMWH was prescribed in all cases of cesarean delivery; since the early postcesarean hospital discharge policy (third day following cesarean) began, we reconsidered whether to continue the prophylaxis after the hospital discharge or not.

Some authors have recommended the postcesarean use of LMWH only when there is an additional risk factor or in cases of emergency cesarean [29]. However, we agree with the recommendations of several clinical practice guidelines [9, 22], which have recommended that the threshold for prescribing thromboprophylaxis should be lower in the postnatal period than that in the antenatal period since the risk of developing VTE per day is higher and the duration of exposure shorter. Even some of them [9] recommend that all women, who have had an emergency caesarean section or an elective cesarean section and have one or more additional risk factors for VTE, should receive thromboprophylaxis with LMWH for seven days.

We prescribe LMWH in all cesareans, both elective and emergency, because we cannot know *a priori* whether another thrombosis risk factor will appear in puerperium (e.g., anaemia, infection, hypertension) or whether a longer rest period will be required (e.g., for postpuncture headache, infection). In fact, 98.53% of the present series of women with cesarean had at least one more risk factor.

At our hospital, the high rate of VTE risk factors may be due to the continual and exhaustive surveillance of these VTE risk factors, included period puerperium; so you might be able to detect more risk factors than other hospitals. In our department, a blood test is performed within 24–36 hours after a cesarean section to count red blood cells, leukocytes, and platelets, which detects anaemia that otherwise may go unnoticed.

In our view, the benefits of postcesarean LMWH administration in minimizing the risk of thromboembolic events greatly outweigh the low level of risk associated with its use [33, 34].

In our study, we have not had any adverse effects from the administration of LMWH (bleeding, thrombocytopenia induced by LMWH).

We agree with the recommendations of recent clinical practice guidelines [16]; and although, a cost-effectiveness analysis was not completed specifically for this population subgroup, the cost-effectiveness model for medical patients indicates that prophylaxis with LMWH is cost effective for patients at increased risk. In our investigation, bemiparin 3500 once daily during five days has been sufficient to prevent thromboembolic events, so health costs are lower (two days less than recommended in the clinical practice guideline) [9], although clotting factors may take a few more days to normalize.

## 4. Conclusions

A reduction in the incidence of thromboembolic disease can be achieved by surveillance of risk factors during the pregnancy, delivery, and puerperium alongside physical measures and thromboprophylaxis with low-molecular-weight heparin.

In case of women with cesarean delivery, we recommend adding thromboprophylaxis with low-molecular-weight heparin (like Bemiparin) during five days following cesarean.

These actions seem to be adequate to avoid thromboembolic events, although the continuation of thromboprophylaxis should be considered if other risk factors emerge.

## Figures and Tables

**Table 1:** Characteristics of patients in the study groups and predelivery thrombosis risk factors.

Thromboprophylactic	Group A	Group B
regimen	Bemiparin 3500 IU/5 days	Bemiparin 3500 IU/10 days
Number of cases (*n*)	311	335
Mean age ± SD	31.37 ± 5.24	31.06 ± 5.62
Age ≥35 years (*n*) (%)	89 (28.61%)	98 (29.25%)
Parity (*n*) (%)		
0	149 (47.9%)	174 (51.9%)
1	90 (28.9%)	101 (30.1%)
2	46 (14.8%)	41 (12.2%)
3	13 (4.2%)	13 (3.9%)
≥4	13 (4.2%)	6 (1.8%)
BMI (*n*) (%)		
BMI > 30	142 (45.8%)	116 (34.62%)
BMI > 35	36 (11.6%)	22 (6.56%)
Smoking (*n*) (%)	51 (16.5%)	40 (11.9%)
>10 cigs./day	16 (16%)	9 (2.25%)
Drug consumption (*n*) (%)	1 (0.3%)	1 (0.3%)
Multiple pregnancy (*n*) (%)	13 (4.2%)	19 (5.6%)
Hypertension (*n*) (%)		
Chronic hypertension	6 (1.9%)	0
PIH in previous pregnancy	4 (1.3%)	6 (1.8%)
PIH in current pregnancy	34 (10.9%)	35 (10.4%)
Diabetes (*n*) (%)	15 (4.82%)	26 (1.8%)
Type I	3 (1%)	2 (0.6%)
Gestational diabetes	8 (2.6%)	18 (5.4%)
Carbohydrate intolerance	4 (1.3%)	6 (1.8%)
Previous heart disease (*n*) (%)	0	1 (0.3%)
Immobility (*n*) (%)	0	1 (0.3%)

BMI: body mass index, PIH: pregnancy-induced hypertension.

**Table 2:** Characteristics of the surgery by study group and thrombosis risk factors during the cesarean.

Thromboprophylactic	Group A	Group B
regimen	Bemiparin 3500 IU/5 days	Bemiparin 3500 IU/10 days
(number of cases)	(*n* = 311)	(*n* = 335)
Type of cesarean (*n*) (%)		
Elective	82 (26.4%)	88 (26.4%)
Emergency	229 (73.6%)	247 (73.6%)
Type of anesthesia (*n*) (%)		
Locoregional	278 (89.7%)	290 (86.6%)
General	31 (10.3%)	45 (13.77%)
Delivery GW (mean ± SD)	38.54 ± 2.47	38.66 ± 2.23
Preterm (*n*) (%)	40 (12.9%)	41 (12.4%)
Placental abruption (*n*) (%)	7 (2.3%)	7 (2.1%)
IUGR (*n*) (%)	24 (7.7%)	31 (9.3%)

IUGR: intrauterine growth retardation, GW: weeks of gestation.

**Table 3:** Postcesarean thrombosis risk factors.

Thromboprophylactic regimen	Group A	Group B
(number of cases)	Bemiparin 3500 IU/5 days	Bemiparin 3500 IU/10 days
	(*n* = 311)	(*n* = 335)
Postcesarean anemia (*n*) (%)		
Hb < 11	222 (71.38%)	249 (74.32%)
Hb < 10	161 (51.8%)	179 (53.5%)
Hb ≤ 9	72 (23.4%)	89 (26.6%)
Infection	21 (6.8%)	20 (6%)
Seroma	17 (5.5%)	16 (4.8%)
Postcesarean hypertension	15 (5.7%)	9 (4%)

Hb: hemoglobin.
